# Efficacy of cefiderocol and levofloxacin against *Stenotrophomonas maltophilia* in a hemorrhagic pneumonia mouse model

**DOI:** 10.1128/aac.00944-25

**Published:** 2026-01-30

**Authors:** Waki Imoto, Junko Abe, Norihiro Sakurai, Kengo Kawamoto, Koichi Yamada, Yukihiro Kaneko, Hiroshi Kakeya

**Affiliations:** 1 Department of Infection Control Science, Osaka Metropolitan University Graduate School of Medicine, Osaka, Japan; 2 Department of Infectious Disease Medicine, Osaka Metropolitan University Hospital 543829, Osaka, Japan; 3 Department of Infection Control and Prevention, Osaka Metropolitan University Hospital 543829, Osaka, Japan; 4 Research Center for Infectious Disease Sciences (RCIDS), Osaka Metropolitan University Graduate School of Medicine, Osaka, Japan; 5 Osaka International Research Center for Infectious Diseases (OIRCID), Osaka Metropolitan University, Osaka, Japan; 6 Department of Bacteriology, Osaka Metropolitan University Graduate School of Medicine, Osaka, Japan; Columbia University Irving Medical Center, New York, New York, USA

**Keywords:** cefiderocol, hemorrhagic pneumonia, levofloxacin, *Stenotrophomonas maltophilia*, mouse model

## Abstract

Cefiderocol (CFDC) is a promising drug for treating infections caused by *Stenotrophomonas maltophilia*, a multidrug-resistant pathogen. However, no evidence of its efficacy against *S. maltophilia*-induced hemorrhagic pneumonia has been reported. We compared the effects of CFDC and levofloxacin (LVFX) on improving survival as well as reducing bacterial load and pathological effects in a mouse model of *S. maltophilia* hemorrhagic pneumonia. The dosage of LVFX and CFDC was determined using a previously described method to establish *S. maltophilia-*induced hemorrhagic pneumonia in mice. CFDC and LVFX were administered intraperitoneally 3 h after bacterial infection. Treatment was administered using the calculated dosages. CFDC significantly improved survival, showed a tendency to reduce bacterial load in the blood and lungs, and revealed that hemorrhage was suppressed in pathological analysis compared with the control group. These results support the current recommendation for CFDC as a treatment option for *S. maltophilia* infections. However, its efficacy in our model was lower than that of LVFX. This may be due to the superior pulmonary tissue penetration of LVFX. Treatment strategies that consider tissue penetration are necessary for severe *S. maltophilia* infections. However, the use of LVFX must consider the risk of resistance development, whereas CFDC may be advantageous for treating resistant pathogens.

## INTRODUCTION

*Stenotrophomonas maltophilia* is a non-fermenting, gram-negative rod and an opportunistic pathogen with inherently low pathogenicity (1). *S. maltophilia* mainly causes bacteremia and pneumonia (1–4) and rarely causes eye infections (5, 6), endocarditis (7, 8), meningitis (9, 10), gastrointestinal infections (11, 12), liver abscesses (13), urinary tract infections (14, 15), and bone and soft tissue infections (16, 17). In addition, it causes severe pneumonia called “hemorrhagic pneumonia” in highly immunosuppressed patients, such as those who have undergone hematopoietic stem cell transplantation. The mortality rate of hemorrhagic pneumonia caused by *S. maltophilia* has been reported to be nearly 100% (1, 18–21). Effective treatment options for *S. maltophilia* infections are extremely limited, and *S. maltophilia* is resistant to most beta-lactam antibiotics. Under these circumstances, cefiderocol (CFDC), a new antibiotic categorized as a siderophore cephalosporin, is active against *S. maltophilia* and is expected to become a new treatment option, as stated in the treatment guidelines issued by the Infectious Diseases Society of America (22). However, evidence supporting its effectiveness in cases of severe *S. maltophilia* infection is insufficient, and its effectiveness against hemorrhagic pneumonia caused by *S. maltophilia* is unknown. Therefore, we investigated the efficacy of CDFC in a previously established mouse model of *S. maltophilia-*induced hemorrhagic pneumonia, using levofloxacin (LVFX) as the control drug (21).

## MATERIALS AND METHODS

### Bacterial strains

As in a previous study, *S. maltophilia* was clinically isolated from the blood culture of a patient with hematological malignancy and was designated OMU_SM_001 in accordance with our institutional rules (21). The minimum inhibitory concentration (MIC) values of CFDC, LVFX, minocycline (MINO), and sulfamethoxazole/trimethoprim (SMX/TMP) for this strain are listed in Table 1.

**TABLE 1:** MIC values of antibiotics for *S. maltophilia* OMU_SM_001 strain^*a*^

Antibiotic	MIC (μg/mL)
CFDC	0.125
LVFX	1.0
MINO	1.0
TMP/SMX	≤9.5/0.5

^*a*^ CFDC, cefiderocol; LVFX, levofloxacin; MINO, minocycline; SMX, sulfamethoxazole; TMP, trimethoprim.

### Antibiotic susceptibility testing

The MIC of LVFX (Tokyo Chemical Industry, Tokyo, Japan) was determined using E-test (bioMérieux Japan, Tokyo, Japan) according to the method described by the manufacturer, as well as the trace liquid dilution method and the broth microdilution method. The MIC of the CFDC was determined by the broth microdilution method using iron-depleted cation-adjusted Mueller–Hinton (MH) broth provided by Shionogi & Co., Ltd. (Osaka, Japan) (23). The MICs of MINO (Tokyo Chemical Industry, Tokyo, Japan) and SMX/TMP (Tokyo Chemical Industry, Tokyo, Japan) were determined using the broth microdilution method. Quality control of the microdilution method was verified using *Pseudomonas aeruginosa* ATCC 27853, in accordance with Clinical and Laboratory Standard Institute guidelines (24, 25).

### Laboratory animals

Six-week-old female specific pathogen-free ICR mice weighing approximately 20–30 g (Japan SLC, Shizuoka, Japan) were housed in a specific pathogen-free environment at the Laboratory Animal Center, Graduate School of Medicine, Osaka Metropolitan University. The animal housing environment at our institution was maintained at a temperature of 23 ± 3°C with a relative humidity of 50%–60%, and a 12-h light/dark cycle (lights on from 08:00 to 20:00 and off from 20:00 to 08:00). Baseline health assessments of the mice included confirmation that their body weight was within 25–30 g and that there were no abnormalities in food and water intake. All experimental procedures were performed according to the guidelines for the management and use of experimental animals proposed by the Japan Physiological Society and the United States National Institutes of Health.

### Hemorrhagic pneumonia model

The *S. maltophilia* hemorrhagic pneumonia model was developed using a previously published method (21). *S. maltophilia* was grown overnight on MHII agar, and a single colony was cultured in MHII broth for 24 h. Fifty microliters of this overnight culture were inoculated into 5 mL of MHII broth and incubated at 37°C for 4 h to reach mid-log phase. After centrifugation, the pellet was resuspended in phosphate-buffered saline (PBS) to ~1.5 × 10^9^ CFU/mL. On Day 0, 50 μL of the suspension was administered intratracheally to mice via a sterile 24-Fr plastic catheter (Nipro, Osaka, Japan) under isoflurane inhalation anesthesia with supine position, and isoflurane was adjusted within 2%–5% with a flow rate of 0.5–4 L/min, while monitoring for loss of reflexes and respiration. For immunosuppression, cyclophosphamide (150 mg/kg; Tokyo Chemical Industry, Tokyo, Japan) was administered intraperitoneally 4 and 1 days before inoculation.

### Pharmacokinetics of LVFX and CFDC in the hemorrhagic pneumonia mouse model

#### Collection of serum samples for PK evaluation

The dosages of LVFX and CFDC were determined experimentally using an *S. maltophilia*-induced hemorrhagic pneumonia mouse model prepared according to a previously described method (21). LVFX (Tokyo Chemical Industry, Tokyo, Japan) and CFDC (Shionogi & Co. Ltd., Osaka, Japan) were individually dissolved in saline immediately before use.

CFDC (10 and 100 mg/kg) and LVFX (10 and 100 mg/kg) were administered intraperitoneally 3 h after bacterial infection. At 5, 15, 30, 60, 120, 240, and 360 min after administration of the therapeutic drugs, the mice were anesthetized by isoflurane inhalation, and cardiac blood was collected. In humans, LVFX is administered at 250–750 mg once daily, whereas CFDC is administered at 750–1500 mg two to three times daily. Accordingly, the per-dose amounts corresponded to approximately 10 mg/kg for LVFX and 10–20 mg/kg for CFDC. Based on this, a dose of 10 mg/kg was selected for both agents to approximate human dosing. In addition, since mice have a faster drug metabolism than humans, a higher dose was also set at 100 mg/kg, which represents the maximum amount that can be dissolved in 0.4 mL for intraperitoneal administration. Cardiac blood was centrifuged, the supernatant was collected, and serum was obtained. Similarly, to compare the serum and lung concentrations, CFDC and LFVX were administered intraperitoneally to mice with *S. maltophilia* hemorrhagic pneumonia 3 h after infection. At 15, 30, 60, 120, 240, and 360 min after drug injection, the mice were anesthetized by isoflurane inhalation, cardiac blood was collected, and the lungs were harvested. Cardiac blood was centrifuged, the supernatant was collected, and the serum was obtained. The harvested lungs were suspended in 1.0 mL PBS, homogenized using a cell strainer (Corning, New York, USA), and the supernatant was collected by centrifugation.

#### Reagents

To improve the accuracy of the analysis of CFDC and LVFX, the internal standard method was used, in which a fixed amount of an internal standard was added to an unknown sample and a standard sample. It uses ceftazidime as an internal standard for CFDC and moxifloxacin hydrochloride monohydrate as an internal standard for LVFX.

Lyophilized CFDC powder was provided as sodium salt by Shionogi & Co., Ltd. (Osaka, Japan). Ceftazidime (the internal standard for CFDC), LVFX, moxifloxacin hydrochloride monohydrate (the internal standard for LVFX), and trifluoroacetic acid were purchased from TCI Co., Ltd. (Tokyo, Japan). Liquid chromatography-mass spectrometry grade acetonitrile and methanol were purchased from FUJIFILM Wako Pure Chemical Corp. (Osaka, Japan). Water was obtained from a Milli-Q purification system (Millipore, Tokyo, Japan).

#### Analytical method

CFDC concentrations in the serum and lung homogenates were determined using liquid chromatography-tandem mass spectrometry (LC-MS/MS). Protein precipitation and ultra-performance liquid chromatography (UPLC) chromatographic conditions were performed using previously reported methods. A 3 µL aliquot of the diluted supernatant was injected onto the LC-MS-MS system consisting of UPLC: ACQUITY H-bio (Waters Corp.: Massachusetts, USA) and mass spectrometry: 4000 QTRAP (SCIEX: Massachusetts, USA) using electrospray ionization-positive ionization mode monitoring the production of *m*/*z* 752.221/285 for CFDC and *m*/*z* 547.205/467.9 for ceftazidime (26). LVFX concentrations in the serum and lung homogenates were determined by arranging the UPLC-Fluorescence method using mobile phases A (0.1% trifluoroacetic acid) and B (acetonitrile) (27).

The lower limits of quantification (LLOQ) for both CFDC and LVFX were 0.01 µg/mL. The analytical methods using CFDC and LVFX were validated based on ICH M10 Bioanalytical Method Validation and within-run and between-run precision and accuracy were ≤15% (LLOQ, ≤20%) and 100% ± 15% (LLOQ, within 100% ± 20%), respectively (28). The protein binding ratios of CFDC and LVFX in the mouse serum were 38.7% and 25%, respectively (29, 30).

#### PK analysis

Pharmacokinetic (PK) parameters were calculated based on a non-compartmental model using Microsoft Excel software (Version 2016). The area under the serum concentration-time curve (AUC) was estimated using the trapezoidal method. The lung/serum tissue AUC ratio was calculated by dividing the AUC estimated from the concentration of the lung homogenate with that estimated from the free serum concentration.

### Outcome Measures

The efficacy of CFDC and LVFX was evaluated based on three parameters: (1) improvement in survival outcomes (2), reduction in bacterial burden in cardiac blood and lung homogenates, and (3) histopathological assessment of lung tissue. All experiments were performed at doses determined by the PK analyses.

Ten mice with hemorrhagic pneumonia were treated with each drug (CFDC, LVFX, or saline as a control) in the survival experiments. Saline (control), LVFX (100 mg/kg), or CFDC (100 mg/kg) was administered 3 h after *S. maltophilia* infection and every 6 h thereafter. Survival was evaluated for 7 days. Statistical analysis of survival was performed using the log-rank test, and survival rates were calculated using the Kaplan–Meier method. Correction for multiple comparisons was performed using the Holm method, and *P*-values less than 0.05 were considered significantly different.

Three mice with hemorrhagic pneumonia were treated with each drug (CFDC, LVFX, or saline as a control) to determine the bacterial load in the lungs and cardiac blood. Bacterial loads in the lungs and cardiac blood were determined by sacrificing the mice at 30 h post-infection and comparing the three groups. Cardiac blood was diluted with PBS and spread on MHII agar plates, and the lungs were removed and homogenized in PBS (1 mL) using a cell strainer (Corning). The lung homogenates were diluted and spread onto MHII agar plates. The plates were incubated at 37°C to calculate the bacterial load in cardiac and pulmonary blood. The Kruskal–Wallis test was used to analyze the bacterial load as a log value. Differences were considered statistically significant at *P* < 0.05. If the Kruskal–Wallis test yielded significant differences, the Mann–Whitney *U* test was used, and Holm-corrected *P*-values < 0.05 were considered statistically significant.

Three mice with hemorrhagic pneumonia were used for each drug (CFDC, LVFX, or saline as a control) for the histopathological assessment of lung tissue. Mice with hemorrhagic pneumonia were generated using the same procedure as that used for the bacterial load experiments. Beginning at 3 h after infection, saline, CFDC, or LVFX was administered every 6 h. At 30 h post-infection, the mice were euthanized, and the lungs were harvested, fixed in formalin, and subjected to histopathological examination using hematoxylin and eosin (HE) staining. The lung histopathological findings were compared among the saline, CFDC, and LVFX groups.

All statistical analyses were performed using EZR software (Saitama Medical Center, Jichi Medical University, Saitama, Japan) (31).

## RESULTS

### Experimental *S. maltophilia* strain was susceptible to LVFX and CFDC *in vitro*

The MIC values of LVFX, CFDC, MINO, and SMX/TMP were 1.0 and 0.125, and 1.0 μg/mL and ≤9.5/0.5 μg/mL, respectively (Table 1).

### Serum concentrations and dosage designs of CFDC and LVFX

**Fig 1:**
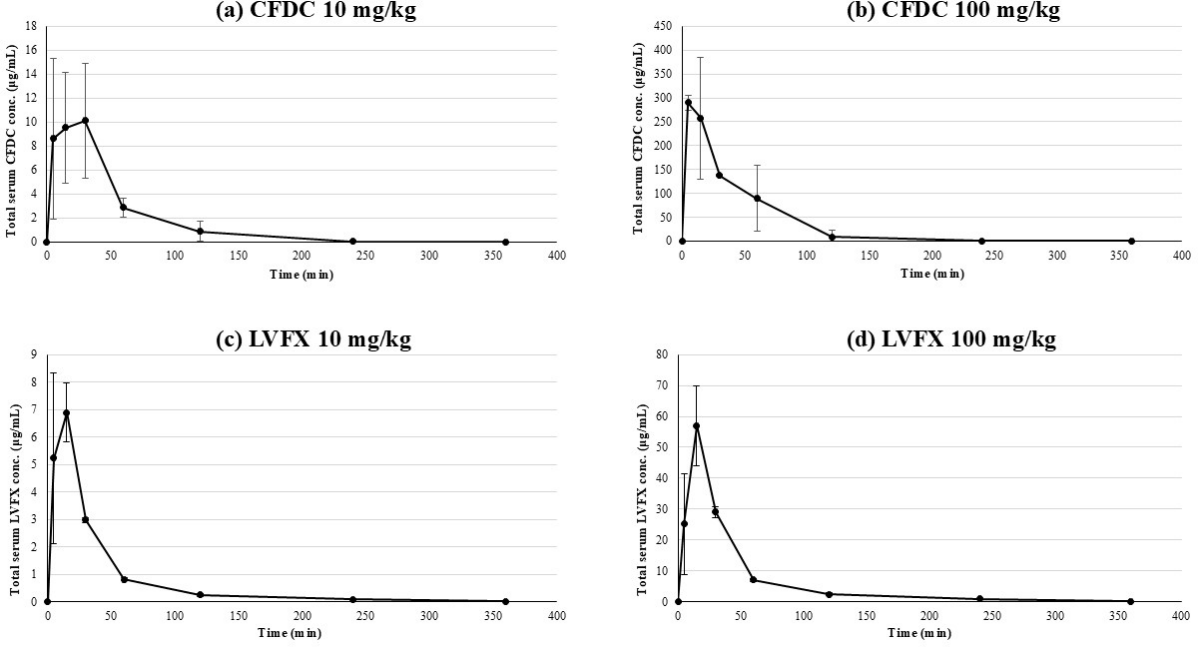
(**a**) Serum concentration of CFDC after intraperitoneal administration of 10 mg/kg. (**b**) Serum concentration of CFDC after intraperitoneal administration of 100 mg/kg. (**c**) Serum concentration of LVFX after intraperitoneal administration of 10 mg/kg. (**d**) Serum concentration of LVFX after intraperitoneal administration of 100 mg/kg.

The serum concentrations of CFDC after administration of 10 and 100 mg/kg of the drug are shown in Fig. 1a and b. When CFDC was administered at a dose of 10 mg/kg, the calculated Cmax and AUC were 10.1 μg/mL and 10.4 μg·h/mL, respectively. At a dose of 100 mg/kg, the Cmax and AUC were calculated to be 290.0 μg/mL and 221.7 μg·h/mL, respectively (Table 2). The protein binding ratio of CFDC in mice has been reported to be 38.7±1.1% (29), and the MIC value measured in this study (0.125 μg/mL) was used to calculate the dose of CFDC such that the time above MIC (TAM) would certainly exceed 54% and be approximately 70% (29). This resulted in a dose of 100 mg/kg every 6 h (TAM was approximately 64%). The serum concentrations of LVFX administered at 10 and 100 mg/kg are shown in Fig. 1c and d. When LVFX was administered at a dose of 10 mg/kg, the calculated Cmax and AUC were 6.9 μg/mL and 4.4 μg·h/mL, respectively. In contrast, at a dose of 100 mg/kg, the calculated Cmax and AUC were 57.1 μg/mL and 37.2 μg·h/mL, respectively (Table 2). The protein binding ratio of LVFX has been reported to be 20%–30% (we used 30% in the calculation) (30), and the dose was calculated to achieve an AUC/MIC ratio >100 using the MIC values determined in this study (1.0 μg/mL). LVFX (100 mg/kg) was administered for 6 h. The detailed serum concentrations and PK profiles of CFDC and LVFX after single intraperitoneal administration are shown in Table 2.

**TABLE 2:** Serum concentrations and pharmacokinetics of CFDC and LVFX following a single intraperitoneal administration^*a*^

Antibiotic	Dose (mg/kg)	Cmax (μg/mL)	T_1/2_ (h)	CL (L/h/kg)	Vd (L/kg)	AUC (μg.h/mL)
CFDC	10	10.1	3.19	0.96	4.43	10.4
	100	290.0	0.33	0.45	0.21	221.7
LVFX	10	6.9	0.99	2.28	3.26	4.4
	100	57.1	1.08	2.69	4.19	37.2

^*a*^ AUC, area under the blood concentration-time curve; CL, clearance; Cmax, maximum blood concentration; Vd, volume of distribution; T_1/2_, elimination half-life.

### CFDC and LVFX improve survival rates in mice with hemorrhagic pneumonia

**Fig 2:**
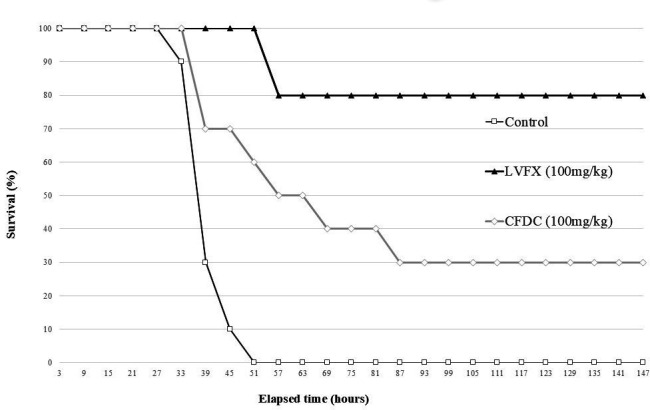
Kaplan–Meier curves for the survival experiment. The open boxes and black lines indicate the control group. Open diamonds and gray lines indicate CFDC groups. The filled triangles and black lines indicate the LVFX group.

The results of the treatment experiments are shown in Fig. 2. All mice in the control group died within 51 h, and survival was significantly higher in the groups treated with CFDC (*P* < 0.01) and LVFX (*P* < 0.01). Survival was significantly improved with LVFX compared to that with CFDC (*P* = 0.021).

### LVFX offers better lung penetration

**Fig 3:**
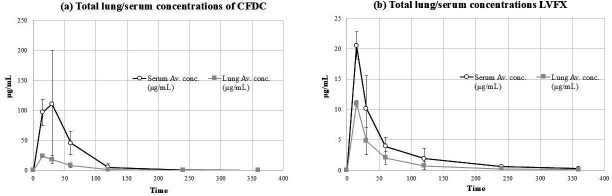
(**a**) Transition of lung and serum CFDC concentrations. (**b**) Transitions of lung and serum LVFX concentrations.

The PK parameters and the lung and total serum concentrations of the CFDC are shown in Table 3 and Fig. 3a. The lung/serum (free concentration) AUC ratio of the CFDC was 0.19. The PK parameters and the lung and total serum concentrations of LVFX are shown in Table 3 and Fig. 3b. LVFX showed a higher lung/serum (free concentration) AUC ratio (0.50) than the CFDC.

**TABLE 3:** Serum and lung concentrations and PK parameters of CFDC and LVFX following a single intraperitoneal administration^*a*^

Antibiotic	Organ	Serum concentration (μg/mL)							AUC (μg.h/mL)
		0 min	15 min	30 min	60 min	120 min	240 min	360 min	
CFDC	Serum	0.0	96.6	110.2	45.4	5.2	0.5	0.1	106.9
	Lung	0.0	23.6	17.9	8.6	1.3	0.0	0.0	21.1
LVFX	Serum	0.0	20.5	10.2	4.0	1.9	0.6	0.3	16.0
	Lung	0.0	11.0	4.8	2.1	0.8	0.3	0.1	8.6

^*a*^ AUC, area under the blood concentration-time curve.

### CFDC and LVFX reduce bacterial burden in the heart and lungs

**Fig 4:**
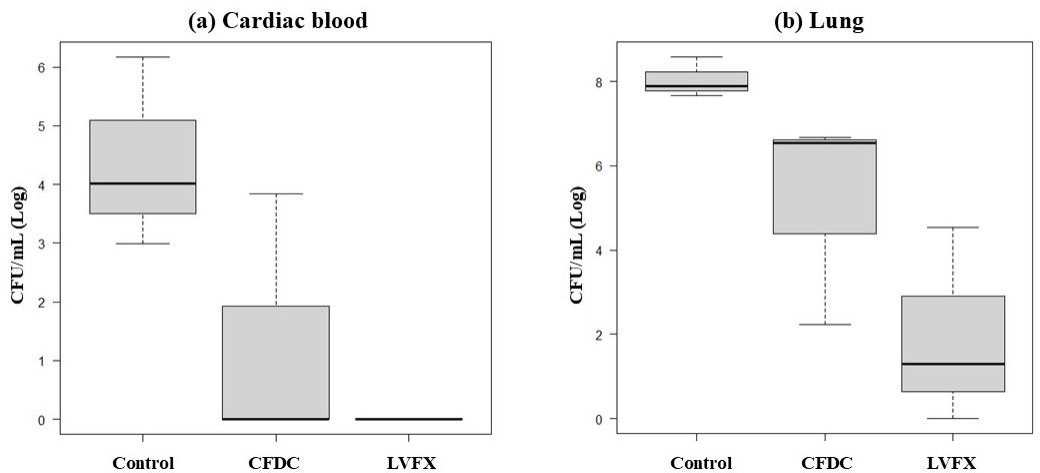
(**a**) Quantitative value of the bacterial load in the cardiac blood (Log_10_ CFU). (**b**) Quantitative value of the bacterial load in the lung homogenate (Log_10_ CFU). Abbreviations: CFDC, cefiderocol; CFU, colony-forming unit; LVFX, levofloxacin.

In the control group, the mean bacterial load in the cardiac blood was 5.0 × 10^5^ CFU/mL, whereas treatment with CFDC reduced it to a mean of 2.3 × 10^3^ CFU/mL, and treatment with LVFX reduced it to below the detection limit (Table 4). Although there was no statistically significant difference, CFDC and LVFX tended to reduce the bacterial load of *S. maltophilia* in cardiac blood, with LVFX showing a greater reduction (*P* = 0.063) (Fig. 4a). Similarly, in the lung homogenates, the mean viable bacterial count was 1.7 × 10^8^ CFU/mL in the control group, whereas it was reduced to a mean of 2.7 × 10^6^ CFU/mL in the CFDC-treated group and 1.1 × 10^4^ CFU/mL in the LVFX-treated group (Table 4). The CFDC and LVFX groups showed lower bacterial load in the lungs than the control group. The Kruskal–Wallis test revealed a significant difference among the groups (*P* = 0.039) (Fig. 4b), and comparison between the groups showed no statistically significant difference owing to the small sample size (control vs CFDC, *P* = 0.30; control vs LVFX, *P* = 0.30; CFDC vs LVFX, *P* = 0.30).

**TABLE 4:** Bacterial burden in cardiac blood and lung homogenates^*a*^

	Study drugs	Viable bacterial count (CFU/mL)	Log-transformed viable bacterial count (Log_10_)
Cardiac blood	Control 1	1.5 × 10^6^	6.18
	Control 2	9.8 × 10^2^	2.99
	Control 3	1.0 × 10^4^	4.01
	Ave. control	5.0 × 10^5^	5.70
	CFDC 1	0	0
	CFDC 2	0	0
	CFDC 3	7.0 × 10^3^	3.85
	Ave. CFDC	2.3 × 10^3^	3.37
	LVFX 1	0	0
	LVFX 2	0	0
	LVFX 3	0	0
	Ave. LVFX	0	0
Lung homogenates	Control 1	3.9 × 10^8^	8.59
	Control 2	4.7 × 10^7^	7.67
	Control 3	7.6 × 10^7^	7.88
	Ave. control	1.7 × 10^8^	8.23
	CFDC 1	1.7 × 10^2^	2.23
	CFDC 2	3.5 × 10^6^	6.54
	CFDC 3	4.7 × 10^6^	6.67
	Ave. CFDC	2.7 × 10^6^	6.44
	LVFX 1	0	0
	LVFX 2	3.4 × 10^4^	4.53
	LVFX 3	2.0 × 10	1.30
	Ave. LVFX	1.1 × 10^4^	4.05

^*a*^ Ave., average; CFDC, cefiderocol; LVFX, levofloxacin.

### CFDC and LVFX improved the lung histopathological findings of hemorrhagic pneumonia

**Fig 5:**
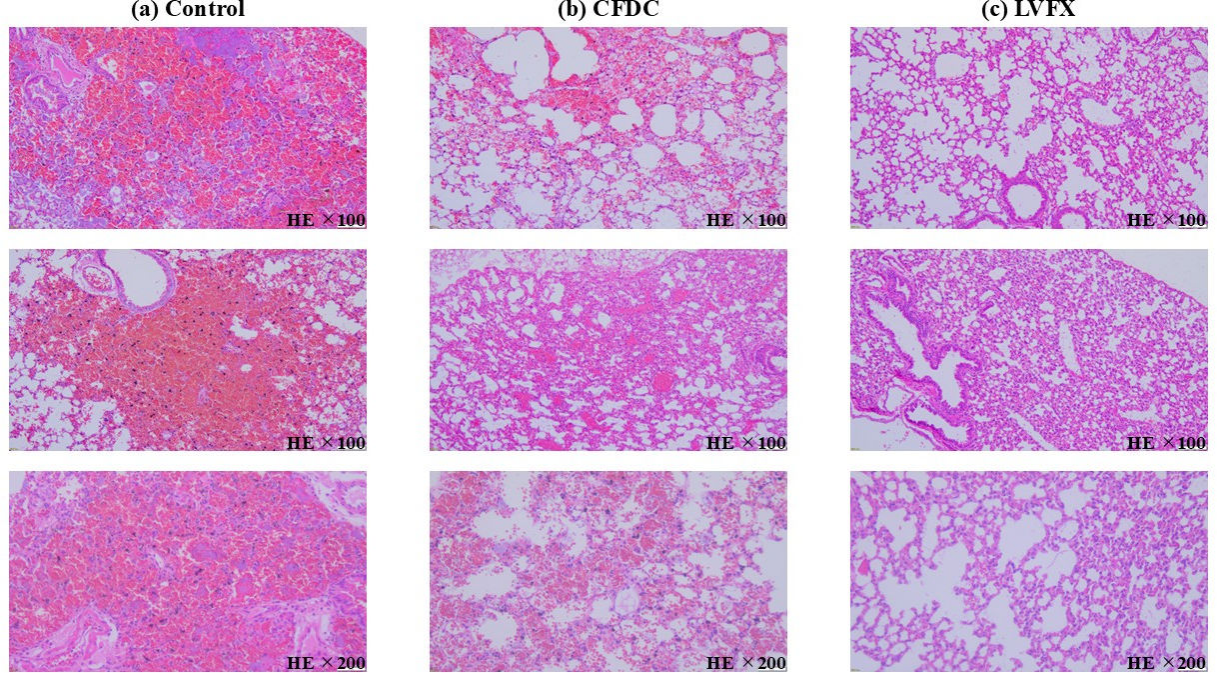
(**a**) Lung histopathology in mice with *S. maltophilia*–induced hemorrhagic pneumonia treated with saline (HE stain; magnification ×100, and 200). (**b**) Lung histopathological findings of *S. maltophilia*–induced hemorrhagic pneumonia mice treated with CFDC (HE stain; magnification ×100, and 200). (**c**) Lung histopathological findings of *S. maltophilia*–induced hemorrhagic pneumonia mice treated with (LVFX) concentrations (HE stain; magnification ×100, and 200).

Pathologically, the degree of hemorrhage was the most severe in control mice, whereas in mice treated with CFDC or LVFX, hemorrhage was suppressed despite the presence of interstitial inflammation. Hemorrhage was rarely observed in mice treated with LVFX; however, slight hemorrhagic manifestations were detected in those receiving CFDC. Therefore, these drugs improved the degree of pathological hemorrhage (Fig. 5a through c).

## DISCUSSION

Both CFDC and LVFX improved the survival rates of mice with *S. maltophilia* hemorrhagic pneumonia, with LVFX showing higher efficacy in this model. To the best of our knowledge, this is the first study to examine the effect of CFDC on *S. maltophilia* hemorrhagic pneumonia and to compare the effects of CFDC and LVFX against *S. maltophilia* in an *in vivo* pneumonia model.

CDFC has been shown to have good activity against *S. maltophilia in vitro* and is recommended in clinical practice for the treatment of mild-to-severe *S. maltophilia* infections (22, 32, 33). Although the effect of *S. maltophilia* on pneumonia has been studied in non-clinical studies using lung infection models in mice and rabbits, its effect has not been proven from model characteristics (34, 35). Additionally, in clinical trials of CFDC that included patients with *S. maltophilia* infection, CFDC was administered to only five patients, four of whom died, and no patients with *S. maltophilia* infection received any control treatment; therefore, the efficacy of CFDC could not be evaluated (36). In this study, mice with *S. maltophilia* hemorrhagic pneumonia that were treated with CFDC showed significantly higher survival rates than control mice. These results align with current clinical recommendations for CFDC use (22).

However, the improvement in survival rate of CFDC-treated mice was lower than that of LVFX-treated mice. In addition, the bacterial load in the blood and lungs tended to be lower in the LVFX group than in the CFDC group, and pathological findings suggested that the degree of hemorrhage was more suppressed with LVFX than with CFDC. Hence, the lung/serum (free concentration) AUC ratio in mice was lower for CFDC than for LVFX, which may explain the observed differences in efficacy in our pulmonary infection model. These results were supported by an experiment comparing bacterial loads in the lungs. Studies in humans have reported that the AUC ratios of the epithelial lining fluid to the serum-free concentration were 0.239 and 1.59–2.69 for CFDC with 2,000 mg and LVFX 500–1,000 mg (37–40). These data for both CFDC and LVFX were obtained from human studies, and in each case, bronchoalveolar lavage fluid concentrations were compared with serum concentrations; therefore, they cannot be directly compared with our study (37–40). However, even in these human studies, LVFX appeared to have superior pulmonary penetration compared with CFDC. Taken together with our findings, this suggests that regardless of the animal species, LVFX is likely to have an advantage over CFDC in terms of tissue penetration into the lungs. Our results suggest that LVFX is an effective treatment option for *S. maltophilia* pneumonia in this mouse model. Conversely, antibiotics must be properly used (41–44), and quinolones are not preferred for all pulmonary infections because *S. maltophilia* may be prone to quinolone resistance. However, in fatal cases, such as hemorrhagic pneumonia, antibiotics with superior penetration may be required with higher priority. In addition, because our model was rapidly fatal, with all mice in the control group dying within 51 h, it is necessary to consider the possibility that quinolones may have been advantageous. Previous guidelines and statements regarding *S. maltophilia* infection do not mention the use of different antibiotics depending on the infected organ (22, 45–47). In the future, treatment strategies that consider tissue migration to the infection focus will be necessary, especially in severe cases, such as hemorrhagic pneumonia.

This study has some limitations. First, this was a non-clinical study conducted in mice. Therefore, these results cannot be directly applied to humans. Secondly, we were unable to compare the efficacy of these drugs with that of TMP/SMX, which has historically been the most commonly used drug for the treatment of *S. maltophilia*. In mice, high blood concentrations of thymidine inactivate TMP/SMX, thereby lowering its effectiveness (48). In the future, establishing hemorrhagic pneumonia models in other animal species and comparing them with TMP/SMX combination drugs will be needed. Next, based on the feasibility of the experiment, the CFDC dosage was set to the amount that achieved a TAM in the 54%–70% range. Although previous studies have shown that this value is sufficient to achieve a therapeutic effect against gram-negative bacilli, it is desirable to achieve a TAM > 100% to recreate the clinical situation. In mice, where drug metabolism is more rapid than in humans, it is more difficult for CFDC, a β-lactam antibiotic for which TAM serves as the pharmacodynamic index, to achieve sufficient efficacy compared with LVFX, a quinolone for which AUC/MIC or Cmax/MIC are the pharmacodynamic indices. It should be noted that due to the relatively rapid metabolism of both agents in mice, the dosing regimens employed were at levels that would not be feasible in humans. Additionally, while previous studies have administered antibiotics subcutaneously to mice, our experiments used intraperitoneal administration (29, 34). This route of antibiotic administration may have resulted in a faster absorption rate than in previous studies, which may have contributed to the lower AUC. Therefore, it is necessary to consider the possibility that the effects of CFDC may have been underestimated. In addition, we were unable to examine whether resistant strains emerged during treatment, nor were we able to investigate whether the PK parameters of each drug in a non-infected model differed from those in the present infection-induced hemorrhagic pneumonia model.

In our preclinical study, LVFX and CFDC demonstrated efficacy both *in vitro* and *in vivo* in a mouse model of *S. maltophilia* hemorrhagic pneumonia, although LVFX showed higher survival rates in this study. Importantly, although LVFX remains an effective treatment option when susceptibility is confirmed, its use may be limited owing to the risk of resistance development. Mutations in genes related to iron transport have been suggested to play a role in CFDC resistance (49), and in *S. maltophilia*, mutations in the *tonB–exbB–exbD* region involved in iron acquisition have also been reported to contribute to CFDC resistance (50). However, CFDC has been reported to have low potential for the emergence of resistance under human-simulated exposure (50), show low MIC values, and demonstrate promising *in vitro* results, suggesting that it may offer an advantage in cases involving resistant pathogens. These findings highlight the importance of adequate dose determination based on PK, susceptibility testing, and resistance surveillance to determine optimal antibiotics for effective therapy.

## Data Availability

The data supporting the findings of this study are available from the corresponding author, Waki Imoto, upon reasonable request.
